# Stimulatory and possible antioxidant effects of High Density Green Photons (HDGP) on cellular systems

**Published:** 2014

**Authors:** L Paslaru, A Nastase, L Stefan, R Florea, A Sorop, E Ionescu, I Popescu, S Comorasan

**Affiliations:** * Center of Digestive Diseases and Liver Transplantation, Fundeni Clinical Institute, 022328, Bucharest, Romania; ** University of Medicine and Pharmacy “Carol Davila”, Bucharest, Romania

**Keywords:** antioxidant effects, HDGP, cellular systems

## Abstract

The interactions between the electromagnetic field and the biological systems were extensively investigated, with remarkable results and advanced technologies. Nevertheless, the visible domain of the spectrum has been rather neglected, since the classic physics did not allow electronic transitions induced by visible light. Recently, the interaction of light with the matter has generated a new scientific domain known in Physics as optical manipulation, with the new concepts of optical matter and optical force. This article presents the results of our work concerning in vitro effects of High Density Green Photons (HDGP) irradiation on cell cultures: stimulation of cell proliferation and migration and a possible antioxidant action.

## Introduction

As a fundamental element for life, the living systems developed numerous and different ways to sense, absorb and use the visible light. Recent advances in optobiology have demonstrated that light may be used for modulation and control of the biological function, in the production of biopharmaceuticals and in biomedicine [**1**].

Many studies demonstrated that the effects on cellular systems depend on the illumination parameters (the wavelength of light source, intensity, treatment timing, etc. For a better understanding of these processes, the effects of monochromatic light irradiation were largely studied.

A series of our previous studies revealed that irradiation with High Density Green Photons (HDGP) has complex pleiotropic effects on matter: modification of enzymes kinetics, (2-4) generation of a new local macromolecular architecture with a protective role toward subsequent aggressions, modification of transmembranar transport [**5**-**9**].

The other studies demonstrated even an in ovo cell proliferation under green light irradiations [**10**,**11**]. Various other effects of green light laser irradiation on different substrates were also described [**12**,**13**].

The purpose of the present paper is to reveal an alternative, and maybe new aspects concerning the irradiations of different cell cultures with high-density-green-photons (HDGP).

## Material and methods

**Cell cultures**

MEF and HUH7 cell line were cultivated in Dulbecco’s modified Eagle’ medium (DMEM) (Gibco, Life Technologies) supplemented with 10% heat-inactivated fetal bovine serum (FBS) and 1% antibiotic. Cells were seeded at different densities, in 3.5 cm Petri dishes, or 24 well plates, and incubated at 37˚C under a humidified atmosphere, with 5% CO2.

**Oxidative stress induction: Cell cultures treatment with hydrogen peroxide**

For the oxidative stress induction, cells were treated with different concentrations of Hydrogen peroxide in the presence (simultaneously or consecutive) or absence of green light irradiation for different durations; right after the treatment, the culture medium was changed and cells were incubated at 37˚C. Usually, cells were processed for subsequent experiments after 24,48 or 72 hours of incubation. During this incubation time, supplementary irradiations were performed.

**High-density green photon source and irradiation**

Cell cultures under different conditions were irradiated with a source of HDGP at different time points. As a source of HDGP, a pure monochromatic light emitting diodes (LED) was used (16V, 20W, 1000 lumens, EverRedTronics, E 20 WG 120 C) Diodes were mounted on ventilated copper radiators. A monochromatic green light with an absorption peak centered at λ-520nm was obtained, with intensities up to 140mW/cm2, spectral width of 10 nm. Time of cells irradiation varied between 1-60 min, at different intervals of time (e.g. 2x5 minutes /24hrs of incubation).

**RNA isolation**

The total RNA was prepared from cell lines by using Trireagent (Sigma, St. Louis, MO) according to manufacturer’s instructions. The quantity and quality of the total RNA were assessed by spectrophotometry with Nano Drop 1000 (Thermo Scientific, Arlington, TX). Samples with a ratio 260/280 of 1.8-2.1 were used in the downstream analysis.

**cDNA synthesis and Quantitative PCR (qPCR)**

First cDNA was obtained from 2 μg of the total RNA by using High Capacity cDNA Archive Kit (ABI, Foster City, CA) in a total volume of 20μl. The final dilution of the samples was 2 ng/μl. The two-step relative quantification was performed on 7300 Real time PCR (ABI, Foster City, CA) by using hydrolysis probes. Then qPCR amplification was carried out in triplicate for each sample in a total volume of 25 μl in the following conditions: 95°C for 10 min, 95°C for 15 sec and 1 min at 60°C for 40 cycles. The level of each mRNA was normalized to reference gene hu18S (20x). Fold changes within tumoral tissues were determined compared with paired non-tumoral tissue. Data were analyzed with SDS 1.4 software using comparative Ct method [2^(-delta delta Ct)]. The tested genes were super oxide dismutase (SOD1) and catalase (CAT). As an endogenous control, 18S gene was used.

**Cell proliferation assay**

Control and HDGP irradiated cells were incubated for a different period of time and cell proliferation was measured by two methods: using Alamar blue (Sigma –Aldrich) staining (assay cells were treated with Alamar Blue and after 1-3h incubation, the shift of color was determined spectrophotometrically) or by using the Tali® Image Cytometer (Life Technologies).

**Cell migration assay**

A scratch assay model [**14**] was used to determine cell migration. Briefly, cells were seeded at the same concentration in 3,5 cm Petri dishes. When cells reached confluence, scratches were made in cells monolayer with a sterile pipette tip. Irradiation of cells was performed: 4 times x 5min / 36hrs. At the beginning of the experiment, images were taken and at regular intervals, the closure of the scratch by migrating cells was microscopically monitorized. After 36hrs of incubations, the cells were stained with Coomassie blue and images were taken.

**Catalase activity**

The catalase activity was assessed by Catalase Activity assay kit (BioVision, CA). Samples and positive control were prepared in the same manner. Briefly, cells were homogenized with Assay Buffer, centrifuged at 10000g for 15 min at 4°C. Supernatant was collected for assay. The standard curve was drawn by using different concentrations of H2O2: 0, 2, 4, 6, 8 and 10 nmols.

**Statistical analysis**

All the statistical analyses were performed by using Graph Pad Prism 5.0 (GraphPad Software Inc, San Diego, CA). The comparisons between groups were performed by using nonparametric tests (Mann–Whitney U-test). Differences were considered significant if p-value was <0.05.

## Results

**SOD gene expression is modified by HDGP action**

The results of the qPCR experiments are presented as an average of three experiments conducted in the same conditions. The control sample was used as calibrator for the analysis.

**Fig. 1:**
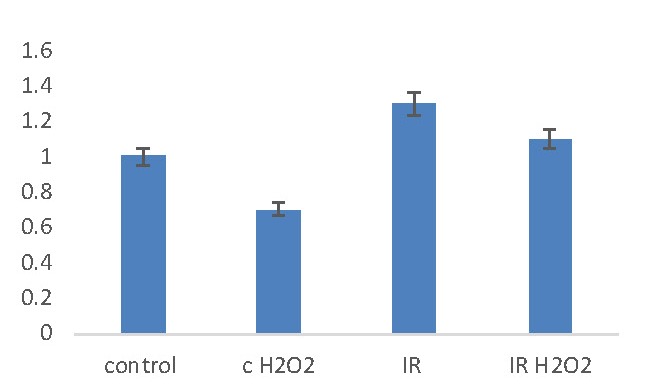
Quantitative polymerase chain reaction measurements of gene expression of SOD in samples with various treatments (H202, irradiated or irradiated in the presence of H2O2) compared with control (no treatment) relative to 18S. The values are expressed as mean of 3 independent replicates

Hydrogen peroxide addition and/ or HDGP irradiation modify cellular SOD gene expression (**Fig. 1**): in the hydrogen peroxide treated cells, the fold change of SOD determined by qPCR decreased compared with the control sample (fold change 0.7, p-value=0.009). The irradiated sample (IR) showed a greater increase in the fold change compared with the control sample (fold change 1.3, p-value=0.03) and also an increase of fold change compared to the hydrogen peroxide treated sample. In the sample which was simultaneously treated with H2O2 and irradiated with HDGP, the SOD gene expression is higher compared with the control sample (fold change 1.1, p-value=0.2), but smaller compared with the irradiated sample and increased compared to hydrogen peroxide sample.

**Catalase activity is modified by HDGP irradiation**

**Fig. 2:**
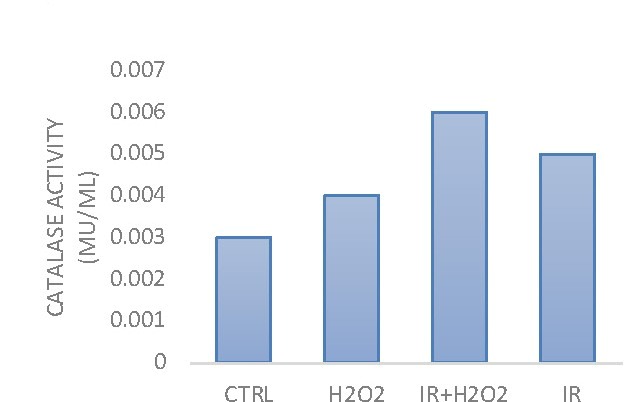
Catalase activity determined by a spectrophotometric test that measured the shift of color at 570nm

Compared with the control sample, the catalase activity was higher in treated hydrogen peroxide and in irradiated samples. In the sample simultaneously treated with H2O2 and irradiated with HDGP, the catalase activity was the highest determined among the studied samples (**Fig. 2**).

**Proliferation assay**

A series of cell proliferation experiments was performed. A stimulation of the cell proliferation was observed in all the experiments; the degree of this stimulation was mainly the function of irradiation protocol. Irradiation for longer period of time (40-60 min) might induce an inhibition of proliferation for certain cell type (data not shown).

**Fig. 3:**
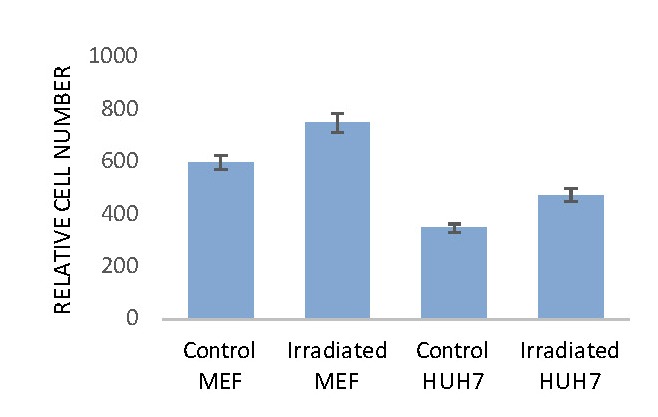
Cell Proliferation assay determined by Tali Image Cytometer. A higher proliferation was observed in MEF cell line compared with HUH7 cell line

The simulative effects of HDGP irradiations (4x5 min/48 hrs incubation) on the proliferation of the two cell lines: MEF and HUH 7 are presented in **Fig. 3**.

**MEFs migration rate is enhanced by exposure to HDGP**

To study the migration capacities of MEFs (controls and exposed to HDGP), a scratch model experiment was used: scratches (”wound gaps”) were made in the cells monolayer and the ”healing” of the gaps by cell migration and growth toward the center was monitored.

**Fig. 4:**
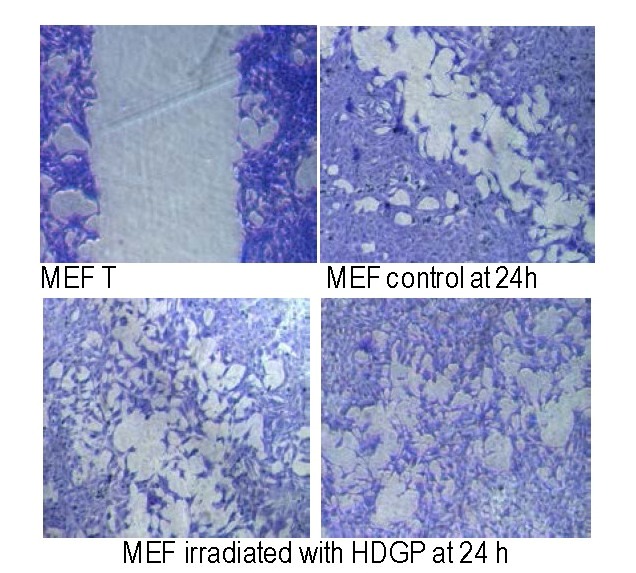
Scratch assay in MEF cell line. For a better visualization of the migration assay, cells were stained with Coomassie blue. Pictures were taken at T0 and after 36h of incubation both for controls and for irradiated samples

The microscope images were taken after 36 hrs. Comparing the images the capacity to close the gaps was evaluated and it was observed that the exposure to green light significantly activated the MEFs migration capacity (**Fig. 4**).

## Discussions and Conclusions

Our previous studies presented a series of new effects of green light irradiations on different substrates [**2**-**9**].

The mechanisms underlying the effects of visible light on cells are incompletely understood.

The present study demonstrated that high-density green photons (HDGP) irradiation stimulates cellular proliferation and migration. Mitochondria could be a possible site for the initial light effects: an increase of ATP production and consequently, stimulation and modulation in levels of growth factors, cytokines and other parameters. In turn, these effects led to an increased cell proliferation and migration with further modulations of cellular processes.

Our results concerning SOD gene expression and catalase activity indicate that HDGP irradiations also induce changes in intracellular antioxidant processes and consequently suggest possible antioxidant effects.

As a conclusion, we consider that all these cellular effects described, indicate that HDGP irradiation may have beneficial bio-medical applications, particularly in the field of regenerative medicine.

**Founding source:** This study was financially supported by the Romanian Program for Research, Development and Innovation, research grant PN ID 139/5.10.2011
